# Association of diet quality with the risk of Sarcopenia based on the Chinese diet balance index 2016: a cross-sectional study among Chinese adults in Henan Province

**DOI:** 10.1186/s12889-023-16933-9

**Published:** 2023-10-17

**Authors:** Xiaodong Ran, Junya Zhai, Minmin Xu, Xiaoxi Zhu, Amin Ullah, Quanjun Lyu

**Affiliations:** 1 Department of Nutrition and Food Hygiene, College of Public Health, Zhengzhou University, Zhengzhou, People’s Republic of China; 2 Department of Clinical Nutrition, The Affiliated Cancer Hospital of Zhengzhou University, Henan Cancer Hospital, Zhengzhou, People’s Republic of China; 3 The First Affiliated Hospital of Zhengzhou University, Zhengzhou, People’s Republic of China; 4 Department of Nutrition and Food Hygiene, College of Public Health, Zhengzhou University and Zhengzhou Shuqing Medical College, Zhengzhou, People’s Republic of China

**Keywords:** Diet quality, Sarcopenia, Chinese Diet Balance Index 2016, Food frequency questionnaire

## Abstract

**Background:**

Sarcopenia can lead to a series of unfavourable health outcomes. Diet is an important factor influencing sarcopenia. In this study, we aimed to evaluate the association of sarcopenia with diet quality assessed by the Chinese Diet Balance Index 2016 (DBI-16).

**Methods:**

A cross-sectional study was conducted to collect information on nutrition and health in Henan Province, China, and a total of 644 individuals were studied. Sarcopenia was defined according to the Asian Working Group for Sarcopenia (AWGS) criteria updated in 2019. Diet quality was assessed by using the Chinese Diet Balance Index 2016 (DBI-16), which includes three indicators: the lower bound score (LBS), higher bound score (HBS) and diet quality distance (DQD). Binary logistic regression analysis was used to estimate the risk of sarcopenia associated with diet quality.

**Results:**

A total of 49 of the 644 participants were diagnosed with sarcopenia. Excessive intake (score > 0) of cereals, meat, eggs and salt, inadequate intake (score < 0) of vegetables, fruits, dairy products, soybeans and low diet variety were commonly seen in both groups of participants. The participants with sarcopenia had a more serious inadequate intake of fruit than those without sarcopenia (*p* < 0.05). The overall LBS, HBS and DQD in both groups were in the interval of low-level problems. Compared with participants with a suitable LBS, those with an unsuitable LBS were more likely to have a low gait speed (OR: 2.58; 95%CI: 1.13–7.04) after multiple adjustments. However, the other two DBI-16 indicators, the HBS and DQD, were not associated with sarcopenia or its related diagnostic variables.

**Conclusion:**

Unfavourable diet quality, mainly referring to inadequate dietary intake in this study, may be a risk factor for low gait speed.

## Background

Sarcopenia is a progressive and generalized skeletal muscle disorder induced by a continuous loss in muscle strength, muscle function, and skeletal muscle mass [1]. Research has found that muscle mass and strength peak around the age of 40 years, and beyond the age of 50 years, muscle mass and muscle strength decrease at a rate of 1–2% and 1.5-5% per year, respectively [2]. According to epidemiological research, the prevalence of sarcopenia ranges from 5.1 to 21.0% among males and 4.1–16.3% among females in Asian countries [3]. In China, these figures are 8.9–13.4% among males and 10.7–15.1% among females [4]. Sarcopenia has been found to be associated with a higher risk of a series of unfavourable health outcomes, including falls, frailty, functional impairments and mortality [5]. Sarcopenia not only affects the patients themselves but also increases the burden on society and the economy. It is reported that the USA government spends 18.5 billion dollars per year for direct health care related to sarcopenia alone [6]. It is of great significance to explore the related risk factors for the prevention and treatment of sarcopenia.

Unhealthy diet is a major risk factor for noncommunicable diseases globally, including type 2 diabetes mellitus, cardiovascular disease, and certain types of cancer [7–9]. A study conducted among USA adults found that, from 1999 to 2016, despite minor improvements in the overall diet quality, important dietary challenges remained such as continued high intake of low-quality carbohydrates and saturated fat [10]. The relationship between diet and muscle health has also received growing attention recently. Although the loss of muscle mass and decline in physical function are considered to be mainly caused by ageing, nutrition status and diet also play important roles in sarcopenia [11].

A series of a priori indices based on dietary recommendations or dietary patterns associated with chronic disease, such as the Healthy Eating Index (HEI), Alternative Healthy Eating Index (AHEI), and Dietary Approaches to Stop Hypertension (DASH) score, have been commonly used to assess the quality of diet [12–14]. The Dietary Balance Index (DBI), established based on the Dietary Guidelines for Chinese Residents published in 2016, is used to assess the overall diet quality in the Chinese population [15]. The DBI-16 consists of three indicators, including the lower bound score (LBS), higher bound score (HBS) and diet quality distance (DQD), which represent inadequate intake, excessive intake and overall diet imbalance, respectively. Compared with other traditional indices, the ability of the DBI-16 to assess diet quality comprehensively and visually is its greatest advantage. The DBI-16 has been used to investigate the relationship between diet quality and the risk of some other chronic diseases, such as diabetes, stroke and hyperlipidaemia [16–18].

Current research on the association of diet and sarcopenia mostly focused on the effects of single nutrients instead of assessing the diet as a whole [19, 20]. So far, the relationship between dietary quality and the risk of sarcopenia has not been fully investigated in Chinese population. In addition, the dietary quality indices used in previous studies may not suit the characteristics of Chinese diet [21]. Accordingly, in this study, we assessed the dietary quality by using DBI-16, which is a more suitable index for Chinese population, and explored the association of dietary quality and the risk of sarcopenia based on a cross-sectional study of general Chinese adults from Henan Province.

## Participants and methods

### Study design and population

The participants in this cross-sectional study included residents aged 25–75 years from three communities in Henan, China. Information on diet, lifestyle and anthropometry was collected from January to November 2020. After excluding 75 individuals with missing data on diet, variables related to sarcopenia diagnosis and other covariates, a total of 644 participants were included in this study.

This study was approved by the Ethical Review Committee, and written informed consent was obtained from all participants (Protocol 2020–KY-066).

### Sarcopenia assessment

Sarcopenia was defined according to the 2019 AWGS diagnostic criteria [3]. Muscle mass was measured by bioelectrical impedance analysis (Inbody 570, Bio Space, Seoul, Korea). All participants changed into paper gowns and were asked to remove all jewellery and other personal effects that could interfere with the examination. The participants were also asked not to exercise or engage in other physical activity before the examination. The height-adjusted appendicular skeletal muscle mass index (ASMI) was defined as appendicular skeletal muscle mass (ASM) divided by height squared. Low muscle mass was defined as an ASMI below 7.0 kg/m^2^ in males and below 5.7 kg/m^2^ in females. Muscle strength was assessed by grip strength, which was measured using a dynamometer (EH101; Camry, Zhongshan, China), and low grip strength was defined as < 28 kg in males and < 18 kg in females. Physical performance was assessed by usual gait speed (m/s) on a 6-m course, and a slow walking speed was defined as a speed slower than 1.0 m/s. Sarcopenia was defined as low muscle mass plus low muscle strength or physical performance, and severe sarcopenia was diagnosed when all three criteria were met [3]. Considering the small sample size of this study, the sarcopenia stage (including severe sarcopenia) and its elements (low muscle mass, muscle strength, and physical performance) were also analysed.

### Dietary data collection

A semiquantitative Food Frequency Questionnaire (FFQ) that included 68 items for foods and beverages was used to collect information on the participants’ dietary intake in the past year. The FFQ used in this study has been validated for reliability and adjusted for the dietary habits of the residents in Henan [22, 23]. The frequencies and portion sizes of each item were determined by means of face-to face interviews with the aid of food models. Oil and condiment consumption was assessed with the total amount of household consumption in one month divided by the number of days in the month and the number of members usually eating at home. The division of food categories and the calculation of energy intake were based on the China Food Composition (National Institute of Nutrition and Food Safety, China CDC).

### Chinese diet balance index 2016

The Chinese DBI-16 is a revised version of the DBI-07 that aims to evaluate the overall diet quality in the Chinese population based on the Dietary Guidelines for Chinese Residents published in 2016 [15]. The DBI-16 consists of 14 subgroups and 8 components with different score ranges: [1] cereals (-12-12); [2] vegetables and fruits (vegetables 0–6, fruits 0–6); [3] dairy and soybeans (dairy − 6 − 0, soybeans − 6 − 0); [4] animal foods (meat − 4–4, fish − 4 − 0, eggs − 4–4); [5] empty energy food (cooking oil 0–6, alcohol 0–6); [6] condiments (sugar 0–6, salt 0–6); [7] diet variety (-12-0); and [8] drinking water (-12-0). A score of 0 means that the food intake meets the recommendation of the dietary guidelines. Negative or positive scores indicate insufficient or excessive food intake, respectively. The DBI-16 further divides food into 12 groups to determine diet variety. The guidelines give different recommendations for food intake according to 11 energy levels, so the scoring of each component is also based on 11 levels of energy intake.

The DBI-16 indicators were calculated with the scores for each component, including the lower bound score (LBS), higher bound score (HBS) and diet quality distance (DQD). The LBS was the absolute value of the sum of all negative scores, indicating insufficient food intake. The HBS was the sum of all positive scores, indicating excessive intake. The DQD was the sum of the absolute value of scores for each component, indicating overall dietary imbalance. The ranges of the LBS, HBS, and DQD in our study were 0–60, 0–44, and 0–84, respectively, due to a lack of data on drinking water intake. A score of 0 represented a suitable dietary intake (no problems), a score less than 20% of the total score represented an almost suitable dietary intake (almost no problems), a score between 20% and 40% of the total score represented a minor intake problem (low-level problem), a score between 40% and 60% of the total score represented a moderate intake problem (moderate-level problem), and a score greater than 60% of the total score represented a severe intake problem (high-level problem).

### Assessment of other variables

Information on demographic characteristics and lifestyles, including sex, age, living area, marital status, education level, smoking status, nap frequency and disease status (whether an individual had other chronic diseases, such as hypertension and diabetes), was collected by a general questionnaire. Height and weight were measured by experienced investigators. Physical activity was measured using the Chinese version of the International Physical Activity Questionnaire (IPAQ). The moderate-vigorous physical activity (MET-h/d, MET, metabolic equivalent of task) was calculated for each participant according to Chinese Guidelines for Chinese Residents [24].

### Statistical analysis

All data analyses were performed using SAS statistical software, version 9.3 (SAS Institute, Cary, NC, USA). A *p* value < 0.05 was considered statistically significant. Continuous variables are described by means and standard deviations (mean ± SD) and were compared by using Student’s t test. Categorical variables are described by frequencies and percentages (n, %) and were compared by using the chi-square test. The association of the indicators of the DBI-16 with the risk of sarcopenia and its elements was assessed using binary logistic regression analysis by odds ratios (ORs) and 95% confidence intervals (CIs). In the analysis, Model 1 was the crude model, and Model 2 was adjusted for potential confounders (sex, age, marital status, disease status, nap frequency and physical activity).

## Results

### Characteristics of the participants

Among the 644 participants, 61.7%(n = 397) were male, and 52.9% (n = 341) were over 60 years old. Of these participants, 86.2% (n = 555) were urban residents, and 58.7% (n = 378) had received a high school level education or higher. A total of 49 (7.6%) participants were diagnosed with sarcopenia based on the 2019 AWGS criteria. Table 1 shows the basic characteristics of the participants according to sarcopenia status. Compared with the group without sarcopenia, the group with sarcopenia was more likely to be over 60 years old, have a lower education level and have lower BMI, ASMI and grip strength values.

**Table 1:** Characteristics of the study participants stratified by sarcopenia

Characteristics	All (n = 644)	Nonsarcopenia (n = 595)	Sarcopenia (n = 49)	*p* Value
Sex				0.121
Male	397(61.7)	367(61.7)	30(61.2)	
Female	247(38.3)	228(38.3)	19(38.8)	
Age				0.017
< 60	303(47.1)	288(48.4)	15(30.6)	
>=60	341(52.9)	307(51.6)	34(69.4)	
Area				0.170
Urban	555(86.2)	512(86.1)	43(87.8)	
Rural	89(13.8)	83(13.9)	6(12.2)	
Marital status				0.197
Married	590(91.6)	544(91.4)	46(93.9)	
Others	54(8.4)	51(8.6)	3(6.1)	
Education level				0.007
No education	16(2.5)	16(2.7)	0(0)	
Middle school and below	250(38.8)	225(37.8)	25(51.0)	
High school and above	378(58.7)	354(59.5)	24(49.0)	
Smoking status				0.089
Yes	345(54.0)	321(53.9)	24	
No	299(46.0)	274(46.1)	25	
Presence of other chronic diseases				0.112
Yes	282(43.8)	262(44.0)	20(40.8)	
No	362(56.2)	333(56.0)	29(59.2)	
BMI^1^	25.37 ± 4.62	25.70 ± 4.61	21.46 ± 2.51	< 0.001
ASMI^2^	7.33 ± 1.08	7.43 ± 1.04	6.12 ± 0.69	< 0.001
Grip strength	31.41 ± 9.47	31.95 ± 9.52	25.78 ± 8.15	< 0.001
Gait speed	0.91 ± 0.20	0.91 ± 0.20	0.88 ± 0.13	0.374
MET^3^	16.47 ± 7.98	16.52 ± 7.75	15.27 ± 10.22	0.407
Nap frequency	4.00 ± 2.62	3.99 ± 2.59	3.81 ± 2.93	0.669

Note: ^1^BMI (body mass index). ^2^ASMI (appendicular skeletal muscle mass index). ^3^MET (metabolic equivalent task). Continuous variables are described by means and standard deviations (means ± SDs) and were compared by using the Student’s t test. Categorical variables are described by frequencies and percentages (n, %) and were compared by using the Chi-square test

### Assessments of diet quality

Table 2 shows the distribution of scores for the DBI-16 components. Excessive intake (score > 0) of cereals, meat, eggs and salt were commonly seen in both groups, with corresponding proportions of 80.3%, 47.8%, 70.7%, and 73.4% in the group without sarcopenia and 85.8%, 49.0%, 63.3%, and 69.4% in the group with sarcopenia. In contrast, both groups had a large proportion, from 59.8 to 100%, of inadequate intake (score < 0) of vegetables, fruits, dairy products, soybeans and low diet variety. For the alcoholic beverage, oil and added sugar categories, most participants in the two groups met the recommended dietary intakes (score = 0), with proportions ranging from 65.4 to 98.0%. Only the fruit category showed significant differences in score distribution between the two groups. The group with sarcopenia had a larger percentage of inadequate fruit intake (*p* < 0.05).

**Table 2:** Distribution of scores for the DBI-16 components and the percentage of participants with each score

Components	Score range^1^	Group	Distribution of scores (n%)													
			(-12)- (-11)	(-10)- (-9)	(-8)- (-7)	(-6)- (-5)	(-4)- (-3)	(-2)- (-1)	0	(1)- (2)	(3)- (4)	(5)- (6)	(7)- (8)	(9)- (10)	(11)- (12)	*p* Value^2^
Cereals	(-12) -(-12)	Nonsarcopenia	0	0	1.0	1.4	2.0	4.7	10.6	8.3	9.8	9.8	10.5	9.5	32.4	0.789
		Sarcopenia	0	0	0	2.0	2.0	2.0	8.2	12.2	18.4	4.1	8.2	17.2	25.7	
Vegetables	(-6) -(0)	Nonsarcopenia				20.6	67.7	10.5	1.2							0.104
		Sarcopenia				28.6	61.2	10.2	0							
Fruits	(-6) -(0)	Nonsarcopenia				19.8	26.5	13.5	40.2							0.014
		Sarcopenia				30.6	14.3	20.4	34.7							
Dairy products	(-6) -(0)	Nonsarcopenia				59.1	29.4	8.1	3.4							0.562
		Sarcopenia				57.1	30.7	12.2	0							
Soybeans	(-6) -(0)	Nonsarcopenia				15.5	35.0	19.1	30.4							0.739
		Sarcopenia				10.2	30.6	24.5	34.7							
Red meats/products, Poultry/game	(-4) -(4)	Nonsarcopenia					2.4	22.3	27.5	23.5	24.3					0.221
		Sarcopenia					8.2	18.4	24.4	18.4	30.6					
Fish/shrimp	(-4) -(0)	Nonsarcopenia					61.1	24.7	14.2							0.924
		Sarcopenia					67.3	18.4	14.3							
Eggs	(-4) -(4)	Nonsarcopenia					4.6	11.0	13.7	22.1	48.6					0.308
		Sarcopenia					10.2	10.2	16.3	16.3	47.0					
Cooking oils	(0)-(6)	Nonsarcopenia							65.4	23.6	6.3	4.7				0.601
		Sarcopenia							75.5	14.3	6.1	4.1				
Alcoholic beverages	(0)-(6)	Nonsarcopenia							85.7	8.6	3.5	2.2				0.740
		Sarcopenia							87.7	8.2	4.1	0				
Added sugar	(0)-(6)	Nonsarcopenia							95.5	1.4	1.2	1.9				0.666
		Sarcopenia							98.0	0	2.0	0				
Salt	(0)-(6)	Nonsarcopenia							26.6	67.1	6.3	0				0.913
		Sarcopenia							30.6	67.4	2.0	0				
Diet variety	(-12) -(0)	Nonsarcopenia	0	2.2	12.8	30.2	36.4	17.6	0.8							0.438
		Sarcopenia	0	4.1	22.4	34.7	24.5	14.3	0							

Note: ^1^Score range of the total score is from − 60 to 44. ^2^*p* value for the chi-square test for the proportions of the scores for each food category

Table 3 shows the distribution of the DBI-16 indicators among the participants. The LBS indicates the degree of inadequate dietary intake; 66.3% and 24.5% of the participants in the group without sarcopenia had low and moderate levels of insufficient intake, respectively. In contrast, 61.2% and 32.7% of the participants in the sarcopenia group had low and moderate inadequate intake levels, respectively. The HBS indicates excessive dietary intake; more than half of the participants had low levels of excessive intake, the percentage of which was 57.4% in the group without sarcopenia and 67.3% in the group with sarcopenia. The DQD was used to evaluate overall dietary intake imbalance, and 53.7% and 44.4% of the participants without sarcopenia had low and moderate levels of imbalanced dietary intake, respectively. The figures were 51.1% and 46.9% in the group with sarcopenia.

**Table 3:** Distribution of the DBI-16 indicators and the percentage of each category

Diet Quality	Indicator	Score Range	Group	Mean ± SD	Distribution of Dietary Quality (n%)^1^				
					No problems	Almost no problems	Low-level problems	Moderate-level problems	High-level problems
Under intake	LBS	0–60	Nonsarcopenia	20.46 ± 5.81	0	8.5	66.3	24.5	0.7
			sarcopenia	21.88 ± 6.21	0	6.1	61.2	32.7	0
Over intake	HBS	0–44	Nonsarcopenia	12.56 ± 5.22	0	30.1	57.4	12.3	0.2
			sarcopenia	11.84 ± 4.36	0	28.6	67.3	4.1	0
Overall imbalance	DQD	0–84	Nonsarcopenia	33.03 ± 7.49	0	1.7	53.7	44.4	0.2
			sarcopenia	33.71 ± 7.28	0	2.0	51.1	46.9	0

Note: ^1^Distribution of the lower bound score (LBS): No problems: 0; Almost no problems: 1–12; Low-level problem: 13–24; Moderate-level problem: 25–36; High-level problem: 37–60. Distribution of the higher bound score (HBS): No problems: 0; Almost no problems: 1–9; Low-level problem: 10–18; Moderate-level problem: 19–27; High-level problem: 28–44. Distribution of the diet quality distance (DQD): No problems: 0; Almost no problems: 1–17; Low-level problem: 18–34; Moderate-level problem: 35–50; High-level problem: 51–84

### Association of the DBI-16 indicators with the risk of Sarcopenia and its components

The results of the binary logistic regression analysis are shown in Table 4. Compared with participants with a suitable LBS, those with an unsuitable LBS were more likely to have a low gait speed (OR: 2.58; 95%CI: 1.13–7.04) after adjusting for sex, age, marital status, disease status, physical activity and nap frequency. The LBS was not found to be related to the risk of sarcopenia, ASMI value or grip strength. In addition, a statistical association was not observed among the HBS, DQD and risk of sarcopenia or its components in the unadjusted and multivariable-adjusted models (Model 1 and Model 2).

**Table 4:** Association of the DBI-16 indicators with the risk of sarcopenia and its elements

	LBS^1^		HBS^2^		DQD^3^	
	Suitable	Unsuitable	Suitable	Unsuitable	Suitable	Unsuitable
Sarcopenia						
Model 1^7^	1	0.71(0.17,2.02)	1	0.93(0.47,1.74)	1	1.21(0.07,6.53)
Model 2^8^	1	0.93(0.22,2.79)	1	1.00(0.49,1.93)	1	1.83(0.10,10.83)
ASMI^4^						
Model 1^7^	1	1.24(0.46,2.82)	1	0.98(0.54,1.72)	1	2.15(0.32,8.59)
Model 2^8^	1	1.49(0.54,3.53)	1	1.03(0.55,1.86)	1	3.04(0.44,13.15)
Grip strength^5^						
Model 1^7^	1	0.50(0.15,1.27)	1	1.01(0.62,1.63)	1	1.47(0.22,5.80)
Model 2^8^	1	0.51(0.15,1.33)	1	0.88(0.51,1.47)	1	1.87(0.27,8.02)
Gait speed^6^						
Model 1^7^	1	2.04(0.96,5.03)	1	1.10(0.74,1.66)	1	2.93(0.55,53.98)
Model 2^8^	1	2.58(1.13,7.04)	1	1.11(0.73,1.71)	1	2.85(0.51,53.39)

Note: ^1^Lower bound score (LBS): Suitable, score of 0 ~ 12; Unsuitable, score of 13 ~ 60. ^2^Higher bound score (HBS): Suitable, score of 0 ~ 9; Unsuitable, score of 10 ~ 44. ^3^Diet quality distance (DQD): suitable, score of 0 ~ 19; unsuitable, score of 20 ~ 84. ^4^ASMI (appendicular skeletal muscle mass index, ASMI < 7.0 kg/m^2^ for males, ASMI < 5.7 kg/m^2^ for females, via BIA). ^5^Grip strength < 28 kg for males, grip strength < 18 kg for females. ^6^Gait speed < 1 m/s. ^7^Model 1: Crude model. ^8^Model 2: Adjusted for sex, age, marital status, disease status, physical activity and nap frequency

## Discussion

In this cross-sectional study conducted in Henan Province, Central China, we observed that diet imbalance was commonly seen in both participants with and without sarcopenia, mainly including inadequate intake of vegetables, fruits, dairy products, soybeans and fish, excessive intake of cereals, eggs and salts and a lack of diet diversity. Moreover, further analyses suggested that a higher LBS, which indicated different degrees of diet intake deficiency, was related to a higher risk of low physical performance after multiple adjustments. However, no relationship was observed between other DBI-16 indicators and the risk of sarcopenia or its components.

Over the past few decades, China has experienced an ongoing transition of dietary patterns, mainly characterized by declines in the intake of cereals and increases in the intake of oils and animal-sourced foods [25]. The results of our study indicated that most participants in the two groups had an excessive intake of cereals, eggs and salt. Cereals remain a major part of Chinese food consumption despite the declining trend [26]. According to the China Nutritional Transition Cohort Study, 77.6% of Chinese individuals aged over 60 years old had consumed more salt than the recommended level [27], which is similar to the result in our study. Studies have shown that overintake of salt could increase the risk of many chronic diseases [28–30]. The results of a study conducted in Japan showed that a high salt diet may lead to fat accumulation and muscle weakness associated with sarcopenia [31]. In our study, no difference in salt consumption between the two groups was observed. In addition, deficient intake of vegetables, fruits, dairy products, soybeans and fish was observed in both groups. A cohort study based on 522 men from the Geelong Osteoporosis Study found that a dietary pattern higher in vegetable, whole grain and animal protein intake was associated with greater skeletal muscle mass over 15 years [32]. According to another prospective cohort study conducted in Hong Kong, a dietary pattern with higher vegetable and fruit intake was associated with lower odds of prevalent sarcopenia in elderly men [33]. In our study, we also found that compared with participants without sarcopenia, those with sarcopenia had lower fruit intake. Dairy, soybeans and fish are all considered to be great sources of premium proteins, which have shown beneficial effects on muscle health [34–36]. No significant difference in the intake of foods in these categories was shown between the two groups in our study, probably because the intake of these foods was low in both groups. Furthermore, it has been reported in some research that food diversity is also connected to the risk of sarcopenia [37, 38].

Several studies have estimated the effects of diet quality on the risk of sarcopenia and its related indicators. A recent study based on the National Health and Nutrition Examination Survey (NHANES), which is a cross-sectional study conducted in the USA, revealed that a higher Healthy Eating Index 2015 (HEI-2015) score, indicating better compliance with the Dietary Guidelines for Americans, might reduce the risk of low grip strength among American adults [39]. Similarly, a study conducted in the elderly Korean population reported that elderly men with low muscle mass and strength had significantly lower recommended food score (RFS) values, which is an indicator of overall diet quality [40]. Moreover, a meta-analysis of 19 studies found that a high adherence to the Mediterranean diet was associated with better walking speed [41]. In our study, we used the DBI-16 to assess diet quality based on the Dietary Guidelines for Chinese Residents and found that a higher LBS, which is one of the DBI-16 indicators representing insufficient dietary intake, was associated with a higher risk of low physical performance defined by gait speed. However, we did not find a link between the other two DBI-16 indicators and the risk of sarcopenia or its components. Consistent with previous studies, our study also showed a trend that a favourable diet quality might improve muscle health status.

The significance of this study was that it was the first study to evaluate the dietary quality by using DBI-16 scores and explore its relationship with sarcopenia in the general Chinese population. The DBI-16 could help people comprehend their dietary imbalance and guide medical departments in establishing prevention and treatment measures quickly and correctly for people at risk of sarcopenia. The primary strength of our study was the use of a dietary assessment index suitable for Chinese people. Compared to the Chinese Healthy Eating Index (CHEI), which is used to estimate adherence to the Dietary Guidelines for Chinese residents [42], the DBI-16 consists of only food indicators without nutrient indicators, which makes its calculation easier and faster. In addition, the DBI-16 could evaluate not only inadequate intake but also excessive intake by calculating the LBS and HBS. Moreover, the DBI-16 scores were based on different energy intake levels for specific individuals, which could control for the confounding effects of total energy intake [43]. Additionally, we defined sarcopenia according to the latest revised criteria of the Asian Working Group for Sarcopenia, which could be better applied in the Chinese population.

There are also some limitations of this study. First, the diet information was collected using a food frequency questionnaire (FFQ), which is a convenient and widely used tool for diet assessment, but it also lacks accuracy and may not be able to fully reflect dietary intake [44]. Second, we calculated the intake of condiments based on the consumption at home but did not take intakes from eating outside of the home into account, which may lead to an underestimation. Third, our study was based on the population of Central China, and the dietary assessment index we used was applicable for the Chinese population, so the findings from the current study should be generalized to other populations with caution. Finally, as a cross-sectional study, our study had a small sample size and was not able to perform an age- or sex-stratified analysis. We plan to continue the current investigation to expand the sample size and carry out follow-up investigations to form a cohort study.

## Conclusion

In this cross-sectional study, we found that excessive intake of cereals, meat, eggs and salt, inadequate intake of vegetables, fruits, dairy products, soybeans and low diet variety were commonly seen in both the sarcopenia and nonsarcopenia groups. The participants with sarcopenia had a more serious inadequate fruit intake than those without sarcopenia. The overall LBS, HBS and DQD in both groups were located in the category of low-level problems. Our results further suggest that unfavourable diet quality, which mainly refers to inadequate dietary intake in this study, may be a risk factor for low gait speed in Chinese residents from Henan Province.

## Data Availability

The datasets used and analysed during the current study are available from the corresponding author upon reasonable request.

## List of abbreviations

DBI: Diet Balance Index
LBS: Lower Bound Score
HBS: Higher Bound Score
DQD: Diet Quality Distance
HEI: Healthy Eating Index
AHEI: Alternative Healthy Eating Index
DASH: Dietary Approaches to Stop Hypertension
FFQ: Food Frequency Questionnaire
BMI: Body Mass Index
ASMI: Appendicular Skeletal Muscle Mass Index
